# A Highly Unstable and Elusive Plasmid That Encodes the Type III Secretion System Is Necessary for Full Virulence in the Marine Fish Pathogen *Photobacterium damselae* subsp. *piscicida*

**DOI:** 10.3390/ijms23094729

**Published:** 2022-04-25

**Authors:** Saqr Abushattal, Ana Vences, Carlos R. Osorio

**Affiliations:** Departamento de Microbioloxía e Parasitoloxía, Instituto de Acuicultura, Universidade de Santiago de Compostela, 15782 Santiago de Compostela, Spain; saqer_shattal@yahoo.com (S.A.); ana.vences@usc.es (A.V.)

**Keywords:** *Photobacterium damselae* subsp. *piscicida*, fish pasteurellosis, T3SS, plasmid, plasmid curing

## Abstract

The marine bacterium *Photobacterium damselae* subsp. *piscicida* (*Pdp*) causes photobacteriosis in fish and important financial losses in aquaculture, but knowledge of its virulence factors is still scarce. We here demonstrate that an unstable plasmid (pPHDPT3) that encodes a type III secretion system (T3SS) is highly prevalent in *Pdp* strains from different geographical origins and fish host species. We found that pPHDPT3 undergoes curing upon in vitro cultivation, and this instability constitutes a generalized feature of pPHDPT3-like plasmids in *Pdp* strains. pPHDPT3 markers were detected in tissues of naturally-infected moribund fish and in the *Pdp* colonies grown directly from the fish tissues but were undetectable in a fraction of the colonies produced upon the first passage of the primeval colonies on agar plates. Notably, cured strains exhibited a marked reduction in virulence for fish, demonstrating that pPHDPT3 is a major virulence factor of *Pdp*. The attempts to stabilize pPHDPT3 by insertion of antibiotic resistance markers by allelic exchange caused an even greater reduction in virulence. We hypothesize that the existence of a high pressure to shed pPHDPT3 plasmid in vitro caused the selection of clones with off-target mutations and gene rearrangements during the process of genetic modification. Collectively, these results show that pPHDPT3 constitutes a novel, hitherto unreported virulence factor of *Pdp* that shows a high instability in vitro and warn that the picture of *Pdp* virulence genes has been historically underestimated, since the loss of the T3SS and other plasmid-borne genes may have occurred systematically in laboratories for decades.

## 1. Introduction

*Photobacterium damselae* subsp. *piscicida* (hereafter dubbed *Pdp*), a bacterium of the family *Vibrionaceae*, was first isolated in 1963 from natural populations of white perch (*Morone americana*) and striped bass (*Morone saxatilis*) affected by an unknown bacterium in the Chesapeake Bay (Maryland, USA) [1]. Since then, *Pdp* has become one of the most devastating pathogens in marine aquaculture, causing a septicaemic disease termed pasteurellosis, pseudotuberculosis, or photobacteriosis, affecting fish species of high commercial value [2,3], but vaccination strategies are far from being satisfactorily effective [4]. Hence, it is necessary to gain a deeper understanding of the underlying mechanisms of *Pdp* pathogenicity. Infections by *Pdp* are characterized by a bacteremia with disseminated tissue necrosis and pronounced cytopathology [5]. The best characterized virulence factor of *Pdp* is AIP56, a systemically disseminated AB-type toxin that induces apoptosis of macrophages and neutrophils [6,7,8] and is secreted through the type II secretion system [9]. AIP56 is encoded within the small virulence plasmid pPHDP10 [6] that has a high prevalence and stability in *Pdp* populations [10]. In addition, many *Pdp* isolates harbor pPHDP70, a ~70 kb plasmid that encodes the functions for synthesis and utilization of siderophore piscibactin [11]. The curing of pPHDP70 under laboratory conditions caused a reduction of virulence in a turbot fish model, but the role of piscibactin as a virulence factor needs additional investigation. A recent study reported that pPHDP70-like plasmids were present in 91 of 103 *Pdp* isolates tested, demonstrating a high prevalence, but not ubiquity in collections of isolates of this pathogen [10].

Despite being a pathogen recognized since 1964 and with a great financial impact on marine aquaculture, sequencing of the *Pdp* genome was not accomplished until 2017, when the genomes of two Spanish, one Japanese and one American isolate were published in different studies [12,13,14]. Recently, the high quality genome sequences of two Australian *Pdp* isolates have been published [15]. These studies clearly showed that *Pdp* genomes contain numerous insertion sequence (IS) elements in a multicopy fashion, accounting for 12–17% of the total genome [12]. As a result of IS proliferation, the *Pdp* genomes exhibit a large number of pseudogenes and of gene deletions, as well as a dramatic loss of biological functions [12] in comparison with its sibling subspecies *Photobacterium damselae* subsp. *damselae* (hereafter dubbed *Pdd*), a generalist and opportunistic pathogen whose genome has not undergone the massive IS expansion of *Pdp* [16]. This process of gene decay in *Pdp* is considered to be associated with a progressive adaptation to a host-dependent lifestyle, and the acquisition of virulence plasmids by horizontal gene transfer is a major driving force in the speciation process that shaped the two subspecies of *P. damselae* [17].

Recently, the presence of genes of the type III secretion system (T3SS) in two strains, PP3 and SNW-8.1, was published for the first time in *Pdp* [18]. The type III secretion system (T3SS) is a needle-like macromolecular apparatus used by several Gram negative pathogenic bacteria to inject effector proteins into the target eukaryotic host cells [19,20]. The effectors manipulate host cell physiology and cause diverse cellular responses and cell damage [19,21]. T3SS genes have been reported in several species of fish pathogenic bacteria [22], and specifically in members of the family *Vibrionaceae* as *Vibrio parahaemolyticus* [23], *Vibrio alginolyticus* [24] and *Vibrio harveyi* [25], among others.

Unexpectedly, a recent study in our laboratory unveiled that the T3SS genes, far from being an exception of some isolates, are highly prevalent in *Pdp* strains from different hosts and geographical origins isolated between 1980 and 2015 [10]. Notably, the same study warned about the spontaneous loss of T3SS genes in *Pdp* strain PP3 during the attempts to genetically modify this strain, and the loss of T3SS gene markers *vscJ*, *vopD* and *vopB* occurred simultaneously with the loss of putative plasmid-borne genes *traC* and *parAB*, suggesting that the T3SS might be encoded within an unstable genetic element. However, the Illumina-based, draft genome sequence available at that time for *Pdp* PP3 was highly fragmented due to the presence of transposase genes in a multicopy fashion, which prevented deciphering the genome context of the T3SS genes [10].

In the present study, the sequencing of the *Pdp* PP3 genome using a PacBio approach has resolved the PP3 genome into two chromosomes and six putative plasmids and unveiled the location of the T3SS within a large plasmid that we dubbed pPHDPT3. This plasmid was highly unstable in culture conditions, its loss occurring as fast as upon a single subculture step on agar plates. In vitro loss of pPHDPT3 markers was demonstrated to constitute a generalized feature of *Pdp* strains isolated from diseased fish. The virulence for fish of spontaneously cured strains was notably reduced in comparison with the stocks of parental strains that contained plasmid-bearing cells. Collectively, this study provides evidence that pPHDPT3 plasmid constitutes a hitherto unreported virulence factor of *Pdp* that is prevalent in *Pdp* populations and is highly unstable in vitro, and warns that the picture of *Pdp* virulence genes has been historically biased and underestimated.

## 2. Results

### 2.1. General Genome Features of Pdp PP3

In a recent study, we found the presence of T3SS-related genes in the draft genome sequences of two *Pdp* strains PP3 and SNW-8.1 obtained by Illumina sequencing [18]. To investigate the genetic context of T3SS genes in the *Pdp* genome, we carried out the genome sequencing of these two *Pdp* strains using a PacBio approach. This resolved the PP3 genome into ten contigs predicted to represent chromosomes I and II and up to six putative plasmids (Table 1). Sequence analysis revealed that the genes for the T3SS are contained within a plasmid of 133,065 bp which was here dubbed pPHDPT3. The remaining contigs corresponded to a pPHDP10-like plasmid, a pPHDP70-like plasmid, a 286-Kb plasmid encoding tetracycline resistance determinants, and two medium-size putative plasmids, one of which was predicted to include three unassembled contigs (Table 1). Surprisingly, the PacBio-derived sequence of strain SNW-8.1 did not contain the sequences of the T3SS and other pPHDPT3 genes (data not shown), as a consequence of pPHDPT3 plasmid instability and curing (see below).

The *Pdp* PP3 chromosomes I and II exhibit sizes (3.1 and 1.1 Mb, respectively) similar to other *Pdp* genomes [12,15]. We identified 644 transposase genes in the PP3 genome, belonging to 18 distinct families. Of note, chromosome II was particularly abundant in the density of IS elements. The distribution of ISs within families and replicons in the PP3 genome is summarized in Table 2.

The pPHDP10 plasmid of PP3 (8115 bb) shared the same structure and similar sequence length with homologous plasmids in two recently sequenced *Pdp* strains from Australia while differing in size and in gene arrangement from the prototype pPHDP10 sequence (9631 bp), first reported in the European *Pdp* strain MT1415 (Supplementary Figure S1). This small plasmid that encodes AIP56 toxin, a major virulence factor of *Pdp*, has undergone insertions of IS elements in its structure, being frequent the successive events of nested integration of transposase genes. The observation that all the pPHDP10 versions maintain a similar backbone of conserved genes that have escaped IS-mediated inactivation, clearly suggests that these conserved genes fulfill important functions for plasmid maintenance and/or for bacterial fitness and virulence.

The pPHDP70 plasmid and its variants, encode the functions for the synthesis and utilization of siderophore piscibactin and has been proposed to constitute a virulence factor of *Pdp* for fish [11]. Initially considered to be a plasmid exclusive to *Pdp* strains from the European continent, it has been detected in strains from Asia [10] and from Australia [15]. The PP3 genome also contains a pPHDP70-like plasmid, whose size (81,347 bp) and gene structure are highly similar to the plasmids of Australian strains and larger than the pPHDP70 plasmid first reported and sequenced in strain DI21 isolated in Spain (68,686 bp) (Supplementary Figure S2).

IS elements are present in the structure of pPHDP70 plasmids (Supplementary Figure S2), but there are conserved modules that have escaped pseudogenization and IS insertion, indicative of their importance in bacterial fitness and in virulence. The hallmark of these conserved modules is the pathogenicity island that contains the piscibactin siderophore genes. Interestingly, the PP3 and the two Australian pPHDP70-like plasmids (which are larger in size than the DI21 version), contain some genes and pseudogenes related to conjugal transfer (as TraF), which are absent from the DI21 plasmid. These scattered, type IV-related, genes might constitute remnants of an ancient version of pPHDP70 that initially contained the elements to be self-conjugative. The process of gene decay, pseudogenization and gene deletion undergone by *Pdp* genomes in its adaptation to a host-dependent lifestyle might have eroded the conjugation machinery of the primeval pPHDP70.

PP3 contains a large plasmid of 286 Kb dubbed here as pPHDP286 (Table 1), that harbors two tetracycline resistance genes, *tetB* and *tetM*. No close homologs of this plasmid have been previously reported in *Pdp*, but similarity searches unveiled that it shares large sequence modules with a putative plasmid sequenced in a *Pdd* isolate (Wu-1) (GenBank Acc. No. CP018299) (Supplementary Figure S3). In accordance with the presence of *tetB* and *tetM* genes, we found that *Pdp* PP3 is resistant to oxytetracycline, an antibiotic widely used in aquaculture, whereas DI21, an extensively studied *Pdp* strain that does not contain these gene markers [12] tested sensitive to this antibiotic (Supplementary Figure S3). Last, two small putative plasmids of ~37 and 23 kb in the PP3 genome, are highly similar to plasmids previously reported in two Australian strains (AS-16-0540-1 and AS-16-0555-7) (Supplementary Figure S4). One of these plasmids is constituted by contig SRHT02000007.1, whereas the second plasmid is predicted to include the three unassembled contigs SRHT02000001.1, SRHT02000002.1 and SRHT02000003.1.

### 2.2. Sequence Analysis of pPHDPT3, a Highly Unstable Plasmid That Encodes the T3SS Genes in Pdp

**Figure 1:**
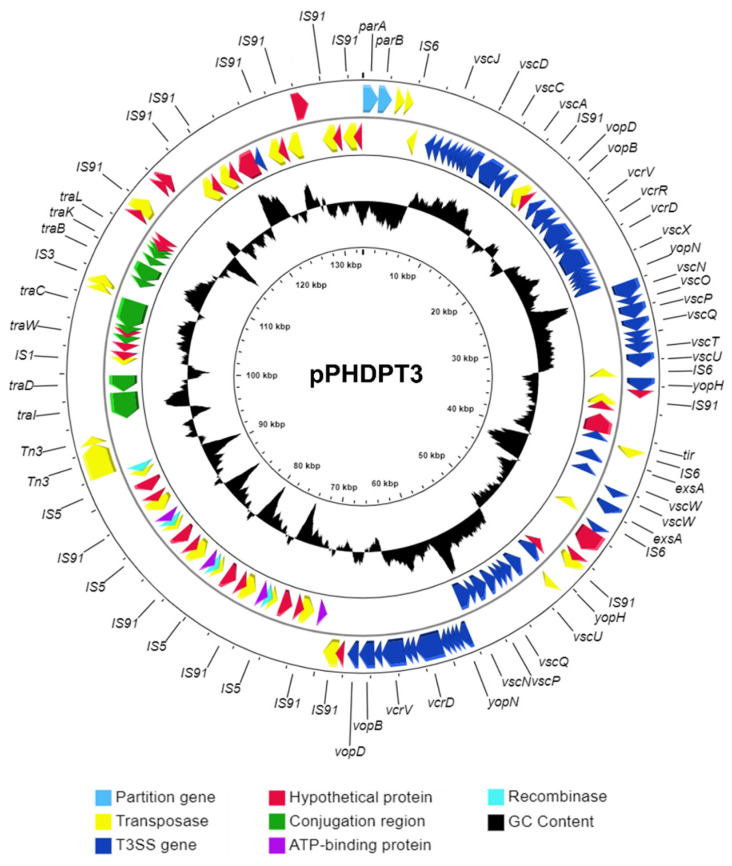
Gene map of pPHDPT3 plasmid of *Pdp* strain PP3 (GenBank accession no. SRHT02000010.1). Arrows represent predicted open reading frames and the orientation of the arrows indicates the direction of transcription for each gene. Map was generated using GCView.

As noted above, genome sequencing of PP3 has unveiled the plasmid location of the genes encoding the T3SS, a potential virulence factor of *Pdp* that has not been functionally characterized so far. The structure and gene organization of the pPHDPT3 sequence obtained with the PacBio approach is shown in Figure 1. pPHDPT3 has an average G+C content of 43%, similar to the host genome (40.9%). A total of 146 coding sequences (CDS) were predicted by the NCBI Prokaryotic Genome Annotation Pipeline, of which 28 were annotated as hypothetical proteins (Supplementary Table S1). Notably, 33 CDS correspond to IS that sum up 29.6 Kb, accounting for 22% of the pPHDPT3 sequence. Six different IS families are present in pPHDPT3, and most of them occur in multicopy (Figure 1; Table 2). IS elements are not uniformly distributed along the plasmid and concentrate in specific regions that separate putative functional modules. The pPHDPT3 IS1 copies have 99% sequence similarity with IS1 of the co-resident plasmids pPHDP10 and pPHDP70, suggesting a high capacity of expansion of this IS, that is present in very high copies in the two *Pdp* chromosomes, as reported in other *Pdp* genomes [12,15]. The pPHDPT3 partitioning system is represented by *parAB* genes.

Notably, sequence analysis did not reveal homology between pPHDPT3 and a known Rep protein, and the analysis of the pPHDPT3 sequence using the DoriC 10.0 database [26] failed to detect replication origin sequences. Last, no sequences were predicted with similarity to the *oriV* of the Marine RNA-based (MRB) plasmid family [27], suggesting that replication of pPHDPT3 may be governed by novel sequences.

**Figure 2:**
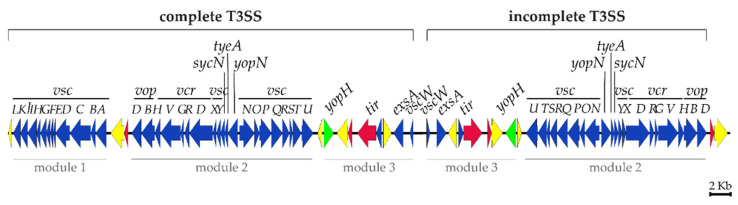
Schematic representation of the complete (loci E4T26_023645- E4T26_023860) and the incomplete (loci E4T26_023865- E4T26_024010) T3SS gene clusters in pPHDPT3 plasmid of *Pdp* strain PP3. T3SS genes (blue arrows) are arranged in three modules separated by transposases (yellow arrows) and hypothetical proteins (red arrows). The gene for the putative effector YopH is highlighted in green.

A prominent feature of pPHDPT3 is the 62,153 bp region that accounts for half of the plasmid sequence and consists of two 100% identical, paralogous copies of T3SS genes organized in opposite orientations, in a mirror-like structure flanked by a transposase gene downstream from *vopBD* genes. The two divergently-oriented gene sets differ by the presence in one of the duplicated sets of a module containing *vscABCDEFGHIJKL* genes in a single copy (Figure 2).

The complete structure of the T3SS includes a basal body that crosses the inner and outer membranes of the bacterial cell, a cytoplasmic sorting platform that plays a role in effector selection and needle creation, and a needle that extends to the extracellular space and is capable of interacting with the host cell membrane [28]. The T3SS gene cluster of pPHDPT3 includes the structural genes of the secretion machinery (Supplementary Table S1) and is divided into three modules separated by transposase genes that do not disrupt any gene function, clearly suggesting the existence of a selective pressure to maintain the T3SS functional. The first module includes *vscABCDEFGHIJKL* genes which are all transcribed from the same strand and are flanked by two different transposase genes. A central, second module includes *vscNOPQRSTUYX*, *yopN*, *tyeA*, *sycN*, *vcrDRGVH*, and *vopBD* genes. A third module contains genes encoding a predicted effector protein YopH-like, a Tir type III secretion system chaperone, a putative transcriptional regulator ExsA, VscW type III secretion system pilotin, transposases and hypothetical proteins (Figure 2). This third module constitutes the symmetry axis point that separates the two paralogous copies of the T3SS genes. The incomplete T3SS gene cluster is 100% identical (at the nucleotide sequence level) to the complete T3SS cluster but lacks *vscABCDEFGHIJKL* genes.

**Figure 3:**
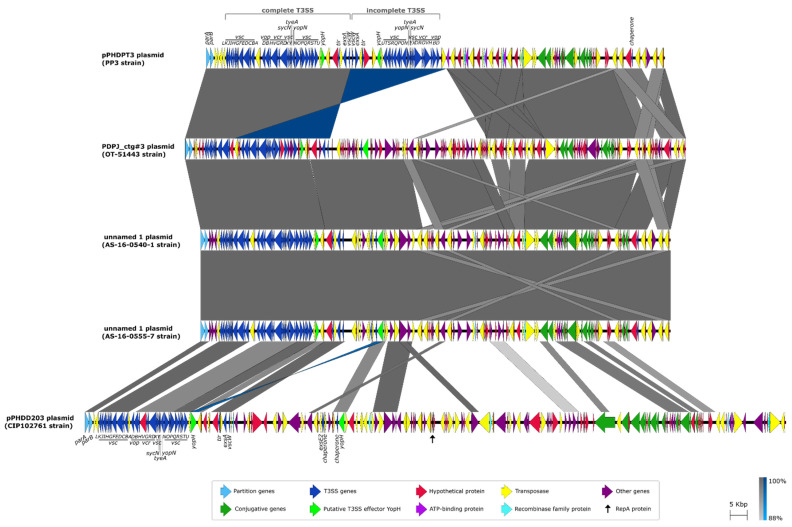
Comparative structural analysis of T3SS-harboring plasmids in four *Pdp* strains: PP3, OT-51443, AS-16-0540-1 and AS-16-0555-7, and in the *Pdd* type strain CIP102761. Grey-shaded and blue vertical blocks between sequences indicate the homologous regions, the color intensity denoting the identity levels (from 94 to 100%).

pPHDPT3 exhibits conserved synteny with plasmids sequenced in the genomes of two Australian (AS-16-0540-1 and AS-16-0555-7) and one Japanese (OT-51443) *Pdp* strain (Figure 3). The similarity was also found with pPHDD203, a plasmid reported in the type strain (CIP102761) of the sibling subspecies *Pdd*, although homology is restricted to the T3SS gene cluster and to scattered plasmid regions (Figure 3). None of these homologous plasmids have been functionally characterized so far [13,15,29]. A comparison of the whole sequences of these five plasmids reveals conserved genetic architecture among them, as well as specific regions (Figure 3).

pPHDPT3 has a 15 kb module of 11 predicted genes *traALEKBVC*, *trbI* and *traWDI* of the conjugative transfer apparatus, and is divided into three submodules by *IS* elements. A comparison of homologous transfer regions in plasmids of the two subspecies shows that the *tra* region in the *Pdp* pPHDPT3-like plasmids is much shorter than in *Pdd* plasmids (Supplementary Figure S5). This comparison provides evidence of the loss of conjugation genes between *traD* and *traW*, represented by the absence of *traGHFNU* and *trbBC* genes in *Pdp* compared to *Pdd*. It is noticeable that the same gene deletions are found in the two Japanese and the two Australian *Pdp* strains. These findings point to the existence of a process of gene decay in pPHDPT3, a plasmid that was likely acquired by conjugation and has subsequently undergone gene deletions during the process of *Pdp* adaptation to a host-dependent lifestyle.

A feature unique to pPHDPT3 of the PP3 strain is the presence of two copies of T3SS genes. In contrast, the PP3 plasmid lacks a large sequence that is otherwise conserved in the other *Pdp* strains. Among other functions, this plasmid region absent in the PP3 plasmid includes a gene encoding an ArdC-like antirestriction protein (locus tag IC628_RS22200 in *Pdp* AS-16-0555-7), that is 89% identical to a protein encoded within the *Pdp* plasmid pPHDP60 (GenBank accession number AGE91731) [30]. Regardless of these sequence divergences among strains, the name of pPHDPT3 is here proposed to name all the *Pdp* plasmids encoding the T3SS that bear similarities among them. It is interesting to note that, in contrast to the four *Pdp* plasmids, the homologous plasmid in the *Pdd* type strain CIP102761 encodes a predicted RepA protein (locus tag VDA_RS23700) (Figure 3).

**Figure 4:**
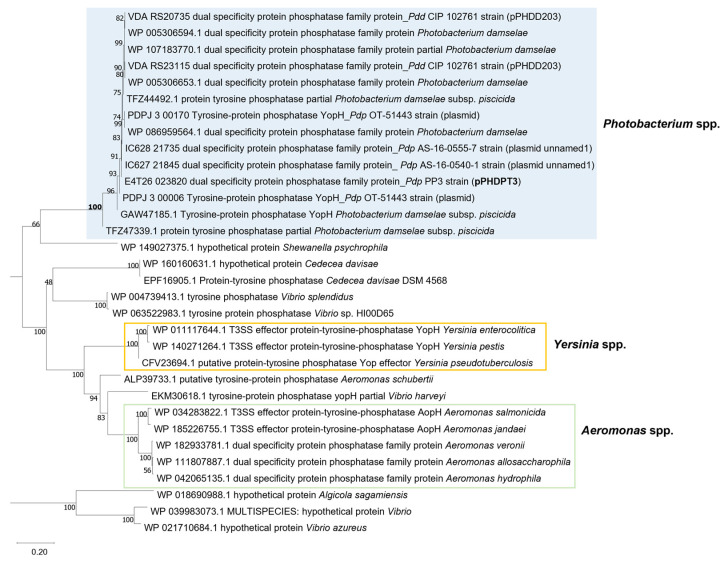
Phylogenetic tree depicting the relationships between *Pdp* and *Pdd* YopH-like tyrosine phosphatases encoded within pPHDPT3-like plasmids, and homologous effector proteins in other bacterial species. The NCBI GenBank accession numbers of the sequences used in the alignment are indicated. Branch length denotes the number of substitutions per site. Bootstrap values are shown next to the branches. Phylogenetic tree was constructed using MEGA-X.

One of the hallmarks of the T3SS reported in other species of the family *Vibrionaceae* is the presence of genes encoding diverse effector proteins that differ considerably among species, and sequence similarity does not always predict accurately their biological roles in the target host cell [31]. Sequence analysis predicts that the *Pdp* PP3 pPHDPT3 sequence encodes a YopH-like putative tyrosine phosphatase protein, that shows similarity to T3SS-dependent effectors in *Aeromonas* spp., *Yersinia* spp. and other genera (Figure 4).

### 2.3. pPHDPT3 Is Highly Unstable and Undergoes Curing upon Cultivation in Laboratory

As indicated above, in this study we conducted a PacBio-based sequencing of the genomes of two *Pdp* strains in which the presence of T3SS genes was first reported, namely PP3 and SNW-8.1. Analysis of the PP3 sequence allowed us to close the sequence of pPHDPT3, the plasmid encoding the T3SS. However, surprisingly, the PacBio sequence files of SNW-8.1 did not contain any sequence related to pPHDPT3 (data not shown), even though the Illumina-based sequence of this same strain conducted in a previous study, unveiled the presence of T3SS gene clusters [18]. In light of the present study, this observation is explained by the random selection of an isolated, plasmid-cured colony of SNW-8.1 as the starting point for genomic DNA extraction for the PacBio sequencing.

**Figure 5:**
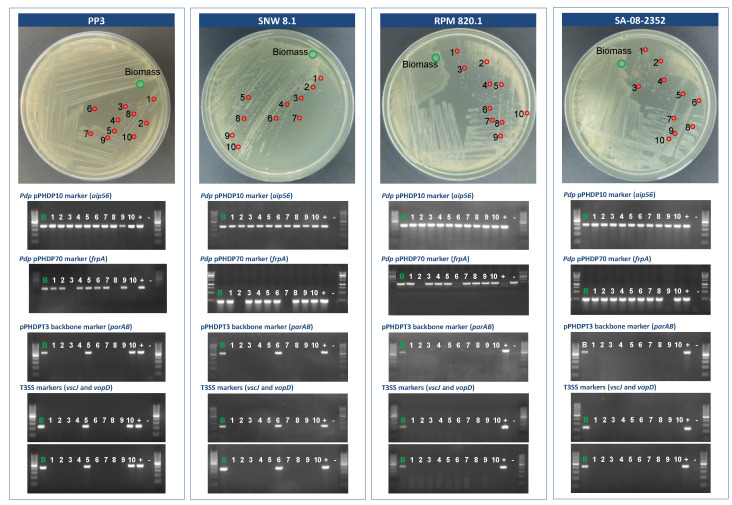
Detection of pPHDPT3 curing in *Pdp* isolated colonies by direct colony-PCR, on agar plates incubated at 25 °C. A colony screening was conducted by PCR amplifications targeted to gene markers of plasmids pPHDP10 (*aip56*), pPHDP70 (*frpA*), and pPHDPT3 (*parAB*, *vscJ*, and *vopD*). Four different *Pdp* strains, PP3, SNW-8.1, RPM820.1 and SA-08-2352 were used. Purified genomic DNA of *Pdp* PP3 was used as positive control. Ten single colonies were randomly selected for each strain to test the stability of pPHDPT3 plasmid plus the cell biomass at the area of confluent growth for each strain. Lane numbers from one to ten denote single colonies of each strain and the biomass is labeled as B-letter. All the chosen colonies yielded a positive result for the *aip56* gene demonstrating the high stability of pPHDP10 plasmid. Evidence of some degree of curing for plasmid pPHDP70 was also detected. The absence of amplification of *parAB*, *vscJ*, and *vopD* markers was indicative of pPHDPT3 curing, and demonstrates the high instability of pPHDPT3 plasmid. Molecular mass ruler of 100 bp is shown at the left and right ends of each gel.

We observed that *Pdp* strains PP3 and SNW-8.1 freshly streaked on a TSA-1 agar plate directly from the frozen stocks, tested positive for T3SS markers on the condition that PCR was conducted using the bacterial biomass of the confluent growth on the first streaks as the source of DNA template for PCR, where it is expected that a fraction of cells still bears the plasmid. However, isolated colonies will be originated from single cells that may or may not harbor the plasmid. Indeed, we found that the majority of the isolated colonies from the same plate tested negative for the T3SS gene markers, indicating pPHDPT3 plasmid loss in the majority of the isolated colonies (Figure 5). Similar results of pPHDPT3 curing in isolated colonies were found in two additional *Pdp* strains of our collection, RPM820.1 and SA-08-2352.

Specific PCR amplifications targeting the *aip56* gene carried on pPHDP10 plasmid and *frpA* gene of pPHDP70 plasmid were conducted in parallel, in order to compare the stability of these two co-resident plasmids with that of pPHDPT3. All the colonies tested positive for pPHDP10 gene markers, corroborating that this plasmid is highly stable in culture conditions. Similarly, most, but not all colonies, tested positive for pPHDP70 markers, indicative of some degree of instability of this plasmid (Figure 5).

The percentage of colonies that lost pPHDPT3 plasmid proved to be similar when bacteria were cultivated at 25 and at 18 °C, suggesting that plasmid curing is not prevented by cultivation at low temperatures (data not shown).

### 2.4. The Instability of pPHDPT3 Is a Generalized Feature of Pdp Isolates: T3SS Genes Are Highly Prevalent in Pdp Isolates and Undergo Loss during Cultivation

Intrigued by this rapid loss of pPHDPT3 genes in *Pdp* isolates maintained in frozen stocks, we wondered whether this is a generalized feature of *Pdp* strains from different sources. In addition, we wanted to assess whether the *Pdp* strains recently isolated from disease outbreaks in fish farms in the European area are positive for pPHDPT3 markers and whether such markers are unstable upon cultivation. To assess this, we analyzed a collection of 51 *Pdp* strains stored at −80 °C in our laboratory. Twenty of them were isolated from diseased European sea bass individuals suffering photobacteriosis in the Atlantic and the Mediterranean basins between 2019 and 2021 and had undergone either one or a maximum of two isolation steps from the primeval TSA agar plates upon fish specimen analysis. The additional thirty-one *Pdp* strains were isolated from diverse sources, between 1980 and 2015. All these strains were streaked from the frozen stocks, and PCR was conducted with the cell biomass in the area of confluent growth. PCR results demonstrated a very high prevalence of the T3SS gene markers *vscJ*, *yopN*, *vopD* and *vscQ* in the cell biomass, as well as of the virulence plasmids pPHDP10 and pPHDP70 (Table 3). In recent strains (isolates from 2014 onwards), the prevalence of plasmid markers was 100%.

We then randomly selected 10 of these strains, representatives of different decades, and performed the same PCR analyses using 10 isolated colonies per strain. As a result, we found that a variable number of isolated colonies tested negative for the four pPHDPT3 plasmid markers simultaneously. Successive passages of those isolated colonies that had yielded positive amplification, caused the eventual loss of the pPHDPT3 markers in all the analyzed colonies, whereas 100% of colonies maintained pPHDP10, and ~80–90% of colonies maintained pPHDP70 (data not shown). These results provide strong evidence that T3SS genes are also encoded in a pPHDPT3-like unstable plasmid in the analyzed *Pdp* isolates, and warn that plasmid curing and, concomitantly, loss of T3SS genes, occur after very few in vitro passages.

We attempted to obtain the sequences of pPHDPT3-like plasmids from two recently isolated *Pdp* strains, using a PacBio approach, with the aim of ascertaining the genetic diversity among this group of elusive plasmids. Even though we selected two *Pdp* strains that had undergone a very reduced number of subculturing passages upon primeval isolation, and despite the final DNA extraction testing positive by PCR for T3SS gene markers, the sequencing results did not contain any sequence related to pPHDPT3, whereas the sequences of chromosome I and II, and of pPHDP10 and pPHDP70 plasmids were resolved with high accuracy (data not shown). These results demonstrate that the ratio of in vitro curing of pPHDPT3 is dramatically fast, and indicates that despite PCR detection of plasmid markers in the DNA sample, the amount of real pPHDPT3-plasmid DNA may not be sufficient for successful sequencing. The reasons for the apparently higher stability of the plasmid in the *Pdp* PP3 strain (stability that allowed for pPHDPT3 sequencing, albeit it also becomes easily cured upon subcultivation) in comparison with other *Pdp* strains are so far unknown.

### 2.5. Tissues of Naturally-Infected Sea Bass Fish Suffering Photobacteriosis Test Positive for pPHDPT3 Genes, and These Become Lost following In Vitro Cultivation

**Figure 6:**
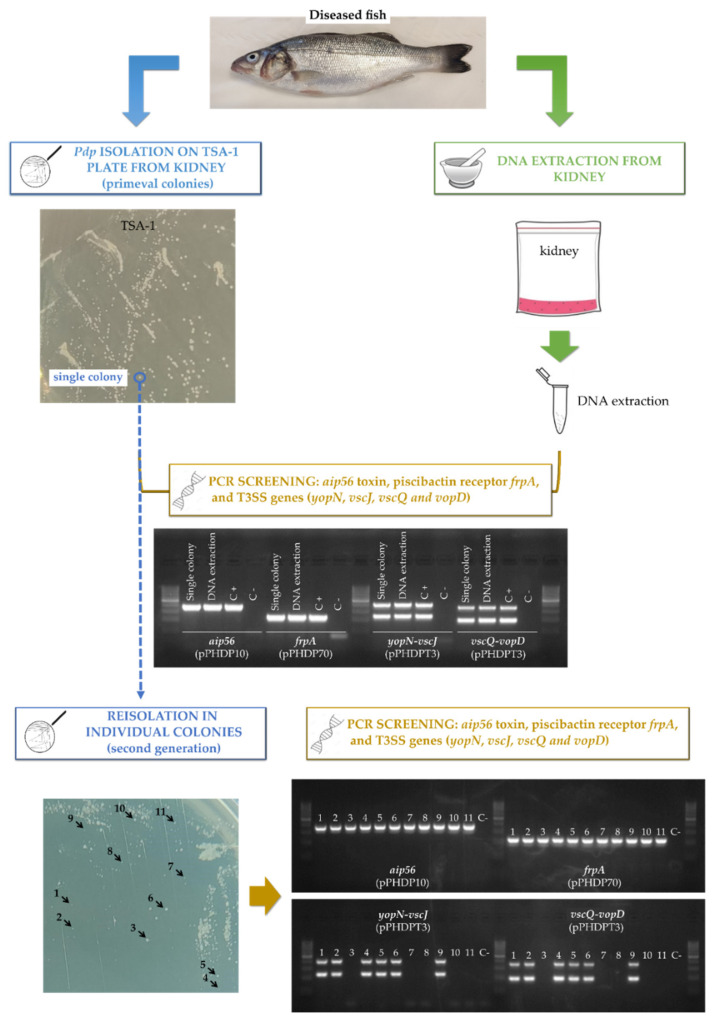
Direct evidence of pPHDPT3 curing upon isolation of *Pdp* colonies from naturally infected European sea bass. Naturally infected fish individuals suffering photobacteriosis were processed in parallel for direct DNA isolation from kidney and for direct plating on TSA-1. Primeval *Pdp* colonies were PCR-assayed for pPHDPT3 markers and selected colonies were re-streaked on TSA-1 to obtain “second generation” colonies. Plasmid markers were detected in DNA from infected kidney as well as in 100% primeval colonies. The PCR analysis of the second generation colonies yielded ~50% colonies negative for all pPHDPT3 markers, indicative of plasmid curing. Numbers 1 to 11 denote eleven “second generation” colonies tested individually. As a control, the markers for the more stable plasmids pPHDP10 and pPHDP70 were amplified in 100% of the second generation colonies.

Up to this point, the laboratory evidence pointing at instability and curing of pPHDPT3 in *Pdp* has been obtained using *Pdp* isolates maintained at −80 °C. In order to assess whether pPHDPT3 presence and further curing can be followed directly from the fish specimens, we analyzed naturally-infected, moribund European sea bass fish from fish farms suffering photobacteriosis outbreaks in field conditions. Immediately upon fish arrival at our laboratory, spleen and kidney samples were taken to purify DNA and to conduct PCR amplification of pPHDPT3 genes and, in parallel, the same specimens were cultivated on TSA-1 plates and incubated at 18 °C until presumptive *Pdp* colonies appeared. Positive amplification was confirmed for pPHDPT3 markers *vscJ*, *yopN*, *vopD* and *vscQ*, as well as for the stable gene marker *aip56* (encoded within pPHDP10) (Figure 6). All the analyzed fish specimens tested positive by PCR for pPHDPT3 markers, and for all the samples we confirmed the growth of *Pdp* colonies after 4–5 days at 18 °C, and these colonies also tested positive for *vscJ*, *yopN*, *vopD* and *vscQ*. When these primeval isolated colonies were subjected to a further streak isolation step, ~50% of the new isolated colonies tested negative for pPHDPT3 markers, indicative of plasmid curing after the first passage on agar plates (Figure 6).

### 2.6. pPHDPT3 Is Essential for Maximal Virulence in Pdp: Virulence for Fish Is Markedly Reduced in Cured Strains

**Figure 7:**
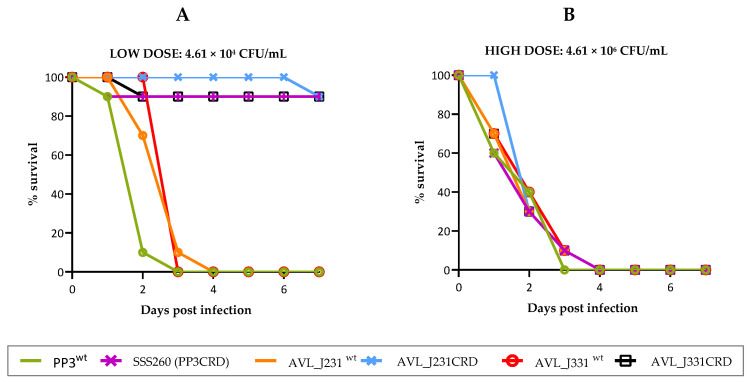
Survival (%) of sea bass fish after intraperitoneal injection of the *P. damselae* subsp. *piscicida* wild type strains PP3, AVL_J231 and AVL_J331 testing positive for pPHDPT3, and their respective cured versions (denoted with a CRD ending). A total of 10 fish were inoculated per strain at two different doses of 4.61 × 10^4^ CFU/fish (**A**), and 4.61 × 10^6^ CFU/fish (**B**). The respective control fish group (10 fish inoculated with 0.1 mL of sterile 0.85% NaCl solution) did not register any mortalities (data not shown).

To assess the role of pPHDPT3 in virulence, we selected an isolated colony of *Pdp* PP3 that underwent spontaneous pPHDPT3 curing and that tested positive for the virulence plasmids pPHDP10 and pPHDP70 (cured strain dubbed SSS260). Bacterial suspensions of SSS260, and of the original parental strain PP3 (bulk cell mass testing positive by PCR for pPHDPT3), were inoculated into European sea bass fish at a sharp dose of ~4.6 × 10^4^ CFU/fish and mortalities were quantified for 7 d. Remarkably, the parental strain killed 100% of fish within the first 4 d post-inoculation, whereas the pPHDPT3-cured strain killed 10% of fish, thus demonstrating a strong impairment of virulence when the inoculated strain is cured of pPHDPT3 plasmid (Figure 7A).

Since we have demonstrated that pPHDPT3 is gradually lost after fish specimens’ cultivation on agar plates and subsequent passages, we wanted to assess if freshly isolated *Pdp* strains directly from diseased fish were more virulent than their isogenic pPHDPT3-cured derivatives. Hence, we inoculated in sea bass fish a dose of ~4.6 × 10^4^ CFU/fish of a *Pdp* strain (AVL_J231) freshly isolated from a moribund fish retrieved from a fish farm undergoing a photobacteriosis outbreak. Inoculation of this strain caused 100% of fish death within 4 d (Figure 7A). In parallel, a single colony of AVL_J231 was picked from the primeval agar plate and subcultured, and a colony that tested negative for pPHDPT3 markers was selected. This pPHDPT3-cured strain (AVL_J231CRD), which proved to test positive for the virulence plasmids pPHDP10 and pPHDP70, only caused death of one fish in the seventh day post-inoculation (Figure 7A). A similar result was obtained when another freshly isolated strain AVL_J331 and its pPHDPT3-cured derivative (AVL_J331CRD) were assayed (Figure 7A). These results altogether provide strong evidence that pPHDPT3 curing impairs virulence of *Pdp* for fish, and warns that plasmid curing may have been occurring for decades in laboratories and causing a gradual loss of virulence of stored *Pdp* strains when subcultured.

Despite the virulence attenuation in pPHDPT3-cured strains shown above, it is well documented that laboratory isolates of *Pdp* do cause fish death when used in experimental inoculations. We, therefore, hypothesized that pPHDPT3-cured strains would maintain a lower degree of virulence compared to plasmid-harboring strains. To gain further insight into this, we conducted fish virulence assays using a dose of ~4.6 × 10^6^ CFU/fish, i.e., 100-fold higher than the previously used dose, with all the strains tested above including their plasmidless derivatives. At this high dose, all the pPHDPT3-cured strains killed 100% of fish within the first 4 d post-inoculation (Figure 7B). These data are in agreement with all the previous studies reporting that *Pdp* strains grown in vitro, are indeed pathogenic for fish in experimental assays. These results shed light on the hypothesis that laboratory strains only cause fish death in experimental infections when high doses are inoculated, while pPHDPT3 plasmid-bearing strains cause fish death at much lower doses.

### 2.7. pPHDPT3 Instability Impedes the Accurate Genetic Manipulation of the Plasmid

In order to dissect the roles of specific pPHDPT3 plasmid genes in the virulence of *Pdp* for fish, we attempted to generate in-frame deletions of selected plasmid genes by allelic exchange. First, we generated constructs to delete the two alleles of *vcrD* gene, encoding one of the structural elements of the T3SS machinery (see methods). We succeeded in selecting a first recombination cross-over that integrated a suicide plasmid of the pGP704 family into pPHDPT3. This integration was possible by selecting Kan^R^, provided by the resistance marker in the suicide plasmid. However, we were unable despite reiterated attempts, to obtain a second recombinant that would eliminate the suicide plasmid. Instead, 100% of the *Pdp* PP3 colonies grown under the conditions that would select for the second recombination cross-over (growth in presence of 15% sucrose without pressure for kanamycin resistance) all tested negative for pPHDPT3 markers (data not shown), indicative of plasmid curing.

We next designed a strategy to force pPHDPT3 stabilization, which included the allelic exchange of each of the two *vcrD* paralogous alleles (*vcrD^1^* and *vcrD^2^*) by a chloramphenicol resistance gene Cm^R^, to generate the two possible *vcrD* single mutants. Next, using the *vcrD^1^* mutant as a starting point, we exchanged the *vcrD^2^* allele with a kanamycin resistance gene Kan^R^, in order to obtain a double mutant labeled with two different resistance genes. By maintaining the presence of the respective antibiotics during all the process of mutant construction, we would expect the allelic exchange to be successful and, at the same time, to stabilize pPHDPT3. As expected, antibiotic pressure eventually allowed the substitution of each *vcrD* allele with a resistance marker (Supplementary Figure S6). This allelic exchange procedure confirmed that indeed the pPHDPT3 plasmid of *Pdp* PP3 contains a duplicated set of T3SS genes. However, we observed that some plasmid gene markers were no longer detected by PCR after the process of mutant construction, indicating that the artificial pressure for maintaining the plasmid, eventually caused the selection of sequence rearrangements and gene loss in pPHDPT3 and, most likely, in the chromosomes (data not shown). In support of this hypothesis of off-target genome alterations caused during mutant construction, we found that the single and double *vcrD* mutants were nonvirulent for fish at the highest dose tested (~4.6 × 10^6^ CFU/fish) (data not shown), i.e., they were much more impaired in virulence than the cured strains. This high instability of the *Pdp* genome, with the presence of IS elements in high copy numbers, and the additional instability of the virulence plasmid pPHDPT3, should warn researchers about the difficulties of generating isogenic mutants for accurate virulence tests with this important pathogen.

## 3. Discussion

After more than 55 years since its first report as a fish pathogen [1], *Pdp* continues to be a major threat to the cultures of many species of fish of importance in marine aquaculture worldwide [32,33,34,35,36,37]. In the past two decades, research has focused on the identification and the study of virulence factors in *Pdp*, the most important so far being the apoptosis-inducing toxin AIP56 encoded within plasmid pPHDP10 [6] and the siderophore piscibactin system encoded within plasmid pPHDP70 [11]. Notably, it was not until 2019 that the presence of genes of the type III secretion system was reported in this pathogen [18]. Soon afterward, it was reported that genes of the T3SS are more the norm than the exception in *Pdp* isolates, and it was suggested that the T3SS might be encoded within an unstable plasmid [10].

A discovery that has been reinforced in recent years is the presence of multiple plasmids in *Pdp* genomes [13,14,15], and indeed the best studied virulence factors are plasmid encoded [6,11]. In the present study, we report that *Pdp* PP3 harbors several plasmids and three of them constitute proved virulence plasmids, pPHDP10, pPHDP70 and pPHDPT3. Despite this clear link between plasmids and virulence in *Pdp*, very little is known so far about the stability of plasmid replicons in *Pdp* cells. pPHDPT3 contains 33 IS of six different families but the T3SS genes are free of *IS* insertions, indicative of selective pressure for maintaining these gene sets intact. We have demonstrated that pPHDPT3 is highly unstable in laboratory culture conditions, and it becomes gradually lost following *Pdp* isolation on agar plates from fish tissues. The reasons for this high instability are so far unknown. Plasmids have evolved a variety of mechanisms that ensure their maintenance after cell division. Changes in the regulation or in the proficiency of these mechanisms caused by in vitro cultivation of bacteria in absence of selection from a fish host, and selective deletions of certain plasmid sequences might alter plasmid stability. Control of plasmid replication and copy number constitutes a major point for plasmid stability [38,39]. Additional strategies include mechanisms for actively partitioning plasmid copies during cell division [40,41], and post-segregational killing mechanisms, such as toxin-antitoxin systems, that selectively kill daughter cells lacking the plasmid [42,43]. In addition, it has to be taken into account that the growth rate can differ substantially between plasmid-free and plasmid-containing cells [44]. Hence, pPHDPT3 plasmid loss might be a consequence of an imperfect functioning of replication/maintenance/partition mechanisms at the molecular level, selective gene deletions occurring during the transition from in vivo to in vitro growth, and/or a consequence of the natural selection that favors the growth of cells that lack the plasmid. pPHDPT3 is a large plasmid that likely constitutes a metabolic burden for replicating cells in vitro, and thus plasmid loss may result in increased fitness outside the fish host. It is evident that *Pdp* cells must have mechanisms to maintain the plasmid in vivo, but not in vitro. We did not find sequences related to plasmid replication in pPHDPT3. Considering the high instability of this plasmid in vitro, and the high content of IS elements, it is not unreasonable to hypothesize that plasmid regions necessary for replication as well as for maintenance might undergo selective deletion and loss upon in vitro cultivation. This hypothesis, which will deserve an in-depth investigation, would explain why this plasmid is so unstable, and why replication-related sequences have not been so far identified in genomic analyses. Selective deletions of sequences necessary for replication and maintenance might also explain the differential stability of pPHDPT3-like plasmids in different *Pdp* strains. This differential stability is exemplified by the presence of enough plasmid DNA in PP3 stocks that enabled PacBio sequencing, in contrast to our inability to sequence homologous plasmids in other *Pdp* strains.

In this line of thought, virulence plasmids that undergo loss and/or sequence rearrangements upon in vitro cultivation have been reported in a variety of bacterial species. A plasmid that encodes nematicidal Cry toxins in *Bacillus thuringiensis* proved to be highly unstable in vitro, with loss occurring within a single growth cycle [45]. Of note, *Aeromonas salmonicida*, a major pathogen of fish in aquaculture, harbors a plasmid encoding the T3SS that is highly prone to undergo genetic rearrangements and T3SS gene loss when *A. salmonicida* is grown at 25 °C, but not when it is grown at 18 °C, and recombination between homologous IS elements has been found to be responsible for deletion and loss of discrete plasmid regions [46,47]. In this study we noticed that pPHDPT3 was unstable during cultivation under laboratory conditions at 25 °C and at 18 °C.

Our observations on plasmid loss in *Pdp* are strongly reminiscent of a similar case very well documented in *Shigella* species. *Shigella* spp. harbor pINV, a ca. 210 kb virulence plasmid containing a pathogenicity island (PAI) of ∼30 kb that encodes a T3SS essential for virulence [48,49]. It was found that plasmid instability differed between *S. flexneri* and *S. sonnei*. pINV is more stable in *S. flexneri*, a species that is mainly transmitted through the environment, and whose pINV variants contain a toxin-antitoxin (TA) system that stabilizes the plasmid [50]. *S. flexneri* can lose the PAI or the entire plasmid during in vitro growth [51]. In contrast, in *S. sonnei*, considered a host-adapted pathogen, pINV has undergone specific, discrete deletions of the genes encoding two TA systems, strongly impairing its stability when grown outside the host at environmental temperatures [52]. This process of TA gene deletions is likely associated with the recent emergence of *S. sonnei* as a host-adapted pathogen. Subsequent studies demonstrated that *S. flexneri* pINV undergoes loss of plasmid regions (including the T3SS) under laboratory conditions, and such deletions occurred through intra-molecular recombination events between ISs flanking T3SS genes [53]. Similarly, *Pdp* is considered to have diverged from its sibling subspecies, *Pdd*, by a process of adaptation to a host-dependent lifestyle, where the high number of IS elements have contributed to gene deletions and pseudogenization that shaped the *Pdp* genome [12]. The presence of large numbers of IS elements confers a high degree of genetic plasticity to pPHDPT3, with the potential to undergo structural rearrangements, fast sequence variations and gene deletions. In support of this idea, we were unable to obtain an isogenic deletion mutant of a pPHDPT3-borne T3SS gene, because the pressure for plasmid maintenance via integration of antibiotic resistance genes into the structure of pPHDPT3 eventually caused the selection of plasmid reorganization events.

The high instability of pPHDPT3-family plasmids, which are highly prevalent in isolates of this pathogen, warns about the fact that its proneness to be lost in cultivation has prevented researchers from detecting and studying these plasmids in *Pdp* isolates worldwide for decades. We demonstrate here that pPHDPT3 gene markers are virtually ubiquitous in all the *Pdp* strains being isolated nowadays from diseased European sea bass in the European area, both in the Atlantic and in the Mediterranean basins. The primeval colonies growing on agar plates upon fish sampling, all tested positive for pPHDPT3 markers. However, laboratory handling and culturing causes the loss of these unstable genetic elements, with special risk when isolated colonies are picked with the intention of establishing new bacterial stocks and starting cultures for research. This has undoubtedly caused an underestimation of the actual virulence gene content of the *Pdp* cells infecting fish in the field. In fact, although pPHDPT3 gene markers are highly prevalent in *Pdp* isolates from diverse geographical origins and isolation sources, the presence of such genes was not discovered and published until as recently as 2019 [18]. Another point that will deserve further investigation, is the differential stability of pPHDPT3 plasmid across *Pdp* strains. In this study, we were able to sequence pPHDPT3 from the genomic DNA of *Pdp* PP3, a strain isolated from *Seriola quinqueradiata* in Japan, that underwent an indeterminate number of passages before it reached our laboratory. Similarly, two recent studies have published *Pdp* genomes that contain a pPHDPT3-like plasmid [13,15] in strains from Japan and Australia. However, we failed to detect pPHDPT3 sequences after a PacBio sequencing of three additional *Pdp* strains. This failing is particularly informative for SNW-8.1, a strain from our laboratory that initially allowed the sequencing of pPHDPT3 pieces by an Illumina strategy [18] but failed to contain the plasmid sequence when a PacBio approach was accomplished later on, and only a single subculture step of the original strain differed between these two sequencing approaches.

The virulence data obtained in this study with plasmid-bearing and with plasmid-cured cultures, clearly demonstrate that plasmid curing causes a strong impairment in the degree of virulence of *Pdp* for fish, albeit pPHDPT3 curing does not completely abolish the pathogenicity of cured strains. These results are in agreement, on the one hand, with the widely reported observations that the *Pdp* strains routinely used for experimental infections do cause fish death and, on the other hand, with the observations that some freshly isolated *Pdp* strains exhibited an unexpectedly higher degree of virulence than usual [54,55]. We hypothesize that the unusually-highly virulent strains reported in some studies correspond to primeval stocks that still preserve the T3SS plasmid in a percentage of cells, whereas the majority of the laboratory strains that have lost the plasmid exhibit higher LD_50_ values. In support of this hypothesis, we were able to detect by PCR the presence of pPHDPT3 markers in a stock of *Pdp* strain PC554.2 that underwent some subculture steps from the stock originally reported as an unusually highly virulent isolate [55], but the genome sequencing of this strain in our present study demonstrated the absence of pPHDPT3 sequences (data not shown). The virulence reduction in *Pdp* stocks that have lost pPHDPT3 plasmid upon isolation and subculturing might explain why some studies have reported a 100-fold difference in LD_50_ among *Pdp* strains isolated in the same case study [56], a difference otherwise very difficult to explain in a pathogen that is highly clonal and serologically homogeneous [55,57,58].

Functions encoded within pPHDPT3 are candidates to play a role in virulence. The *Pdp* T3SS includes a YopH-like tyrosine phosphatase effector, whose homologs in other bacterial species play a major role in virulence, as reported in *Aeromonas salmonicida* [59] and in *Yersinia* spp. [60,61,62]. While the structural genes of T3SS are conserved among related species, effector proteins are highly diverse and some of them are specific for each pathogen. Moreover, fish pathogens are not an exception to this [22]. In this scenario, it is feasible that yet-unknown proteins with effector function and with other roles are encoded in pPHDPT3-like plasmids in different *Pdp* strains.

Recently isolated *Pdp* strains in the European Mediterranean and Atlantic basins test positive for pPHDPT3. Similarly, in a recent study, we reported that genes now known to be pPHDPT3-borne, are highly prevalent in *Pdp* isolates from diverse fish hosts and geographical locations, between 1980 and 2015 [10]. Considering that recently sequenced *Pdp* strains from Japan and Australia also harbor a pPHDPT3-like plasmid [13,15], it is reasonable to propose that pPHDPT3 is a virulence plasmid that is virtually ubiquitous in *Pdp* isolates currently circulating in the world. This high prevalence of pPHDPT3 plasmid and its high instability in vitro, clearly warn that *Pdp* cells in vivo surely exhibit virulence features that have passed unadvertised to microbiologists and fish pathologists since the beginning of *Pdp*-caused outbreaks in aquaculture in the 1960s. Acquisition of the large virulence plasmid pINV encoding a T3SS led to the emergence of *Shigella* spp. from *Escherichia coli* and enabled bacteria to invade epithelial cells [63]. Similarly, it is tempting to speculate that pPHDPT3 might allow *Pdp* to infect fish through ways and/or infective dynamics that have not been so far reproduced under laboratory conditions, considering that plasmid-cured strains have likely been used extensively in experimental infections in laboratories.

## 4. Materials and Methods

### 4.1. Bacteria, Plasmids, and Media

The bacteria and plasmids used in this study are listed in Table 4. *Pdp* strains were routinely grown on tryptic soy agar (TSA-1) or in tryptic soy broth (TSB-1) supplemented with an extra 0.5% NaCl (1% NaCl final concentration) at 25 °C or 18 °C. *Escherichia coli* strains were grown on Luria Bertani agar (LB) plates or Luria Bertani Broth at 37 °C. When necessary, antibiotics were used at the following concentrations: kanamycin (Kan) at 50 µg mL^−1^, ampicillin (Amp) at 50 µg mL^−1^, chloramphenicol (Cm) at 5 µg mL^−1^ or 20 µg mL^−1^. Antimicrobial susceptibility patterns to tetracycline (12 μg per disc) and oxytetracycline (30 μg per disc) were determined by disc diffusion tests on TSA-1 plates, using bacterial suspensions adjusted to an optical density at 600 nm (OD600) of 0.5 in saline solution (0.85% NaCl wt/vol). The diameter (in mm) of the inhibition zones around discs was measured and photographed after 24-h incubation at 25 °C.

### 4.2. DNA Sequencing, Annotation, and Comparative Genomics Analyses

High-purity genomic DNA of *Pdp* strains for genome sequencing was extracted using the G NOME DNA kit (MP Biomedicals) according to the manufacturer’s instructions, and the DNA was subjected to sequencing following the PacBio procedure (SNPsaurus, Eugene, OR, USA). The assembly by Flye 2.4.1 [68] was generated from 1,336,131,874 total bases, for 222 read depth over the genome. The read length N50/N90 was 10795/6571. Coding sequences (CDS) were predicted by the NCBI Prokaryotic Genome Annotation Pipeline [69]. Additional information of gene annotation and gene functions was obtained using the rapid annotations of subsystems technology (RAST) and the BLASTP database [70,71]. The CGView server database was used to obtain a circular graphical map representation of the pPHDPT3 plasmid genome [72]. Comparative analysis of nucleotide sequences was performed using BLASTN and BLASTP databases [71], and MAUVE and EasyFig programs [73,74]. The PacBio-derived sequence of the *Pdp* PP3 genome was deposited in GenBank database under Accession number SRHT00000000.

### 4.3. PCR Amplifications

Primers used in this study are listed in Table 5. These include primers for mutant construction, and for screening of gene presence. Markers related to pPHDPT3 plasmid backbone: *parAB*; genes of the T3SS: *vcrD*, *vscJ*, *vopD*, *vscQ* and *yopN*. PCR primers to test *aip56* gene (pPHDP10 plasmid) and *frpA* genes (pPHDP70 plasmid) were used to screen for the presence of these two virulence plasmids. PCR amplifications were routinely performed using the NZYTaq II 2× Green Master kit following the manufacturer’s instructions; annealing temperatures were adjusted according to the corresponding primer pairs. Colony PCR was routinely conducted by picking an isolated colony and suspending it in 30 µL of sterile distilled water. Three µL of this suspension were directly added to the PCR tube to be used as a DNA template.

### 4.4. Phylogenetic Analysis

The phylogenetic tree of YopH-like putative effectors was constructed in MEGA-X [75]. Protein sequences were aligned using Clustal W [76]. The evolutionary histories were inferred using the Neighbor-Joining method [77]. The percentage of replicate trees in which the associated taxa clustered together in the bootstrap test (1000 replicates) is shown next to the branches. The evolutionary distances were computed using the Poisson Correction method [78], and are in the units of the number of amino acid substitutions per site.

### 4.5. Processing of Diseased Fish Tissues for PCR and Pdp Cultivation

European sea bass (*Dicentrarchus labrax*) fish individuals were obtained from farms located on the Atlantic and the Mediterranean coasts of Spain that were undergoing outbreaks of photobacteriosis caused by *Pdp*. Fish were transported to our laboratory under refrigerated conditions and immediately dissected upon arrival. Kidney and spleen samples were cultured on TSA-1 plates and incubated at 18 °C for 3–4 days until presumptive *Pdp* colonies appeared. Direct colony-PCR was conducted with isolated colonies using appropriate specific primers. In parallel, samples of the kidney and spleen were aseptically removed for total DNA extraction using Easy-DNA^TM^ (Invitrogen), following the manufacturer’s recommendations. Routinely, 200 ng of total DNA was used as a template for PCR reactions to detect the presence of *Pdp* gene markers in the tissues of naturally infected fish.

### 4.6. Selection of pPHDPT3-Cured Strains for Virulence Assays

Parental *Pdp* strains PP3, AVL_J231 and AVL_J331 were streaked on TSA-1 plates and incubated at 18 °C until isolated colonies were observed. Five isolated colonies per strain were re-streaked on fresh TSA-1 plates and incubated at 18 °C. When isolated colonies appeared, between 10–20 colonies per strain were subjected to PCR screening for pPHDPT3 markers (*vscJ*, *vopD*, *yopN*, *vscQ*) as well as for the pPHDP10 plasmid marker *aip56*, and the pPHDP70 marker *frpA*. Colonies that tested negative for all the pPHDPT3 markers but tested positive for *aip56* and *frpA* were considered pPHDPT3-cured colonies and kept for further experiments. This process led to obtaining the cured versions of strains PP3 (dubbed SSS260), AVL_J231 (AVL_J231CRD) and AVL_J331 (AVL_J331CRD) (Table 4).

### 4.7. Virulence Assays

European sea bass fish (*Dicentrarchus labrax*) with an average weight of 5 g were selected to perform virulence tests using three parental *Pdp* strains and their respective pPHDPT3-cured derivatives, as well as three *vcrD* mutant strains (SSS256, SSS299 and SSS325). Fish were acclimated in 100 L aquaria at 24 °C for three days before the injection. For dose preparation, bacterial biomass at the confluent growth zone of the agar plate from 48 h cultures at 18 °C was suspended in saline solution (0.85% NaCl) and adjusted at a sharp OD_600_ = 0.3. Serial decimal dilutions were prepared in saline solution and bacterial counts were carefully calculated. Fish were inoculated intraperitoneally with 0.1 mL of bacterial suspensions in saline solution (0.85% NaCl) using either a low dose (~4.61 × 10^4^ CFU/fish) or a high dose (~4.61 × 10^6^ CFU/fish). A group of 10 fish was inoculated with saline solution as a control. For each strain and dose assayed, 10 fish were inoculated, and mortality was recorded over a period of 7 d after injection. Dead fish were aseptically dissected, bacteria were recovered from the kidney by culturing on TSA-1 agar plates, and colonies were screened by PCR using gene markers for pPHDPT3, pPHDP10 and pPHDP70 plasmids. The protocols of animal experimentation used in this study have been reviewed and approved by the Animal Ethics Committee of the Universidade de Santiago de Compostela, Spain (Ethical approval code 15004/14/003).

### 4.8. Allelic-Exchange Mutagenesis of vcrD Gene Alleles of the T3SS

The coding sequence of each of the two *vcrD* alleles (*vcrD^1^* and *vcrD^2^*) was replaced with antibiotic resistance genes following an allelic exchange procedure using the suicide vector pNidKan, a pCVD442 derivative [66]. Two fragments of ~2 kb, upstream (fragment 1–2) and downstream (fragment 3–4) of each *vcrD* coding sequence were PCR-amplified. Fragment 1–2 was amplified using primers *vcrD*_XhoI_1 and *vcrD*_BamHI_2 while fragment 3–4 was amplified using primers *vcrD*_BamHI_3 and *vcrD*_NotI_4. After being digested with restriction enzymes, the two fragments were ligated so that the resulting DNA sequence generated an in-frame, 1697-bp deletion, of the *vcrD* coding sequence. In order to fix the unstable pPHDPT3 plasmid, the construction containing the deletion was cut with *Bam*HI and an 882 bp chloramphenicol resistance gene (*cat*) amplified from the pKD3 plasmid [67] was inserted. In addition, another version for mutant construction was generated by cutting the aforementioned deletion construction with *Bam*HI and inserting there an 1190 bp PCR-amplified kanamycin resistance gene (*kan*) from the pKD4 plasmid [67]. The *cat*-labelled construction for mutation of *vcrD* alleles was used to obtain single *vcrD^1^* and *vcrD^2^* mutants independently (SSS256 and SSS325 mutant strains, respectively). Besides, in order to obtain a double mutant, the *cat*-labelled *vcrD^1^* mutant was used as the basis to construct a double mutant (SSS299) by using the *kan*-labelled construction that was used to delete *vcrD^2^* gene. The mutant allele containing the *cat* gene was introduced into the suicide vector pNidKan and propagated into *E. coli* β3914. The mutant construction containing the *kan* gene was inserted into the suicide vector pNidKan and propagated into *E. coli* S17-1-λ*pir*. *E. coli* donor strains were conjugated with *Pdp* recipient strains by mixing equal volumes of log-phase cultures of donor and recipient strains on TSA plates prepared with seawater. First recombinants for single *vcrD^1^* mutant construction were selected on TSA-1 plates supplemented with chloramphenicol at 5 µg mL^−1^ (Cm^5^). For double mutant construction, the colonies resulting from the conjugation between *E. coli* S17-1-λ*pir* and *Pdp* PP3 SSS256 *∆vcrD^1^-cat* (single mutant) were selected on TSA-1 plates supplemented with Cm^5^ and kanamycin at 50 µg mL^−1^ (Kan^50^). To select for the second recombination of the single mutant construction containing the *cat* gene, TSA-1 plates supplemented with Cm^5^ and sucrose (15% [wt/vol]) were used. TSA-1 plates supplemented with Cm^5^ + Kan^50^ and sucrose (15% [wt/vol]) were used to select double mutant colonies containing the *kan* gene plus the *cat* gene.

## 5. Conclusions

This study provides evidence that pPHDPT3 plasmid constitutes a novel, hitherto unreported virulence factor of *Pdp*. The high in vitro instability of pPHDPT3-like plasmids warns that the picture of *Pdp* virulence genes has been historically biased and underestimated. The realization of the prevalence of plasmids encoding the T3SS and additional genes in *Pdp* will surely change our view of this pathogen from now on.

## Figures and Tables

**Table 1:** General features of *Pdp* PP3 genome sequenced with the PacBio approach in this study.

PP3 Genome (PacBio)		
GenBank Accession no.	SRHT02000000	
Genome size (bp)	4,918,480	
% GC	40.90%	
Genes (total)	4868	
CDSs (total)	4687	
Contigs	Accession no.	size (bp)
Chromosome I	SRHT02000009.1	3,184,080
Chromosome II	SRHT02000005.1	1,164,185
pPHDP10-like	SRHT02000004.1	8115
pPHDP70-like	SRHT02000008.1	81,347
pPHDPT3	SRHT02000010.1	133,065
pPHDP286	SRHT02000006.1	286,884
plasmid unnamed1	SRHT02000003.1	25,333
plasmid unnamed1	SRHT02000002.1	9318
plasmid unnamed1	SRHT02000001.1	2958
plasmid unnamed2	SRHT02000007.1	23,195

**Table 2:** Distribution of transposases in the major replicons of the *Pdp* PP3 genome sequenced in this study.

IS Family	Chromosome I	Chromosome II	pPHDP10	pPHDP70	pPHDPT3	pPHDP286
IS1-like element ISPda1 family transposase	183	162	0	3	4	0
IS1 family transposase	53	29	0	0	0	0
IS91 family transposase	23	40	3	6	0	1
IS3 family transposase	17	24	0	1	2	1
IS91-like element ISPda2 family transposase	9	14	0	3	15	0
IS4 family transposase	4	7	1	0	0	0
IS4-like element ISVsa5 family transposase	0	0	0	0	0	8
Transposase zinc-binding domain-containing protein	3	3	1	3	0	0
IS30 family transposase	1	0	0	0	0	0
IS6 family transposase	1	0	0	0	6	0
Heteromeric transposase endonuclease subunit TnsA	1	0	0	0	0	0
ISNCY family transposase	1	0	0	0	0	1
IS5/IS1182 family transposase	0	0	0	0	4	0
Tn3 family transposase	0	0	0	0	2	0
Tn3-like element ISKox2 family transposase	0	0	0	0	0	1
IS110 family transposase	0	0	0	0	0	1
IS256 family transposase	0	0	0	0	0	1
Unclassified transposase	0	0	0	1	0	0
Total	296	279	5	17	33	14

**Table 3:** Prevalence of virulence markers of plasmids pPHDP10, pPHDP70 and pPHDPT3 in 51 *Pdp* isolates from disease outbreaks between 1963 and 2021. (+, gene presence; −, gene absence).

Strain	Fish	Location	Year	pPHDP10	pPHDP70	pPHDPT3			
				*aip56*	*frpA*	*vscJ*	*yopN*	*vopD*	*vscQ*
ATCC17911	*Roccus americanus*	USA	1963	+	−	−	−	−	−
MZS8001	*Seriola quinqueradiata*	Japan	1980	−	−	+	+	+	+
EPOY 8803 II	*Epinephelus akaara*	Japan	1988	−	−	−	−	−	−
P3333	*Seriola quinqueradiata*	Japan	1990	+	+	+	+	+	+
DI91	*Sparus aurata*	Spain	1990	+	+	+	+	+	+
B51	*Dicentrarchus labrax*	Spain	1991	+	+	+	+	+	+
ATLIT2	*Morone* sp.	Israel	1993	+	+	−	−	−	−
O69E	*Sparus aurata*	Greece	1994	+	+	+	+	+	+
DS11	*Sparus aurata*	Spain	1995	−	+	−	−	−	−
619.1	*Sparus aurata*	Portugal	1996	+	+	+	+	+	+
LgB53/02	*Solea senegalensis*	Spain	2001	+	−	−	−	−	−
PC609.1	*Sparus aurata*	Spain	2002	+	+	+	+	+	+
TW515/02	*Solea senegalensis*	Spain	2002	+	+	+	+	+	+
PC813.1	*Sparus aurata*	Spain	2003	+	+	+	+	+	+
PC715.1	*Sparus aurata*	Spain	2003	+	+	−	−	−	−
ACC29.1	*Solea senegalensis*	Portugal	2004	+	+	+	+	+	+
ACC71.1	*Solea senegalensis*	Portugal	2006	+	+	+	+	+	+
ACC72.1	*Solea senegalensis*	Portugal	2006	+	+	+	+	+	+
RPM 820.1	*Solea senegalensis*	Spain	2007	+	+	+	+	+	+
AQV6.1	*Solea senegalensis*	Portugal	2007	+	+	+	+	+	+
357/08	*Argyrosomus regius*	Spain	2008	+	+	+	+	+	+
SA-08-2352	*Mugil cephalus*	France	2008	+	+	+	+	+	+
AQV22.1	*Sparus aurata*	Portugal	2008	+	+	+	+	+	+
AQV28.1	*Dicentrarchus labrax*	Portugal	2008	+	+	+	+	+	+
AQP8.1	*Sparus aurata*	Portugal	2009	+	+	+	+	+	+
AQP19.1	*Dicentrarchus labrax*	Portugal	2009	+	+	+	+	+	+
SA082369T	*Scophthalmus maximus*	France	2009	+	−	−	−	−	−
SNW8.1	*Salmo salar*	Spain	2014	+	+	+	+	+	+
SNW13.1	*Salmo salar*	Spain	2014	+	+	+	+	+	+
AQV67.1	*Dicentrarchus labrax*	Portugal	2015	+	+	+	+	+	+
AQV69.1	*Solea senegalensis*	Portugal	2015	+	+	+	+	+	+
Pdp-AVL1902	*Dicentrarchus labrax*	Turkey (Aegean Sea)	2019	+	+	+	+	+	+
Pdp-AVL2001	*Dicentrarchus labrax*	Spain (Mediterranean)	2020	+	+	+	+	+	+
Pdp-AVL2004	*Dicentrarchus labrax*	Spain (Mediterranean)	2020	+	+	+	+	+	+
Pdp-AVL2005	*Dicentrarchus labrax*	Spain (Mediterranean)	2020	+	+	+	+	+	+
Pdp-AVL2006	*Dicentrarchus labrax*	Spain (Atlantic)	2020	+	+	+	+	+	+
Pdp-AVL2011	*Dicentrarchus labrax*	Spain (Atlantic)	2020	+	+	+	+	+	+
Pdp-AVL2012	*Dicentrarchus labrax*	Spain (Atlantic)	2020	+	+	+	+	+	+
Pdp-AVL2017	*Dicentrarchus labrax*	Turkey (Aegean Sea)	2020	+	+	+	+	+	+
Pdp-AVL2101	*Dicentrarchus labrax*	Spain (Mediterranean)	2021	+	+	+	+	+	+
Pdp-AVL2106	*Dicentrarchus labrax*	Spain (Mediterranean)	2021	+	+	+	+	+	+
Pdp-AVL2107	*Dicentrarchus labrax*	Spain (Mediterranean)	2021	+	+	+	+	+	+
Pdp-AVL2109	*Dicentrarchus labrax*	Spain (Mediterranean)	2021	+	+	+	+	+	+
Pdp-AVL2111	*Dicentrarchus labrax*	Spain (Mediterranean)	2021	+	+	+	+	+	+
Pdp-AVL2112	*Dicentrarchus labrax*	Spain (Mediterranean)	2021	+	+	+	+	+	+
Pdp-AVL2113	*Dicentrarchus labrax*	Spain (Atlantic)	2021	+	+	+	+	+	+
Pdp-AVL2114	*Dicentrarchus labrax*	Spain (Atlantic)	2021	+	+	+	+	+	+
Pdp-AVL2115	*Dicentrarchus labrax*	Spain (Atlantic)	2021	+	+	+	+	+	+
Pdp-AVL2122	*Dicentrarchus labrax*	Turkey (Aegean Sea)	2021	+	+	+	+	+	+
Pdp-AVL2134	*Dicentrarchus labrax*	Greece (Aegean Sea)	2021	+	+	+	+	+	+
Pdp-AVL2135	*Dicentrarchus labrax*	Greece (Aegean Sea)	2021	+	+	+	+	+	+

**Table 4:** Bacterial strains and plasmids used in this study.

Strain or Plasmid	Description	Reference/Source
*P. damselae* subsp. *piscicida*		
PP3	Diseased *Seriola quinqueradiata*, Japan	[18]
SNW 8.1	Diseased *Salmo salar*, Spain	[18]
DI21	Diseased *Sparus aurata*, Spain	[57]
RPM 820.1	Diseased *Solea senegalensis*, Spain	[10]
SA-08-2352	Diseased *Mugil cephalus*, France	[10]
AVL_J231	Diseased *Dicentrarchus labrax*, Spain	This study
AVL_J231CRD	AVL_J231 cured of pPHDPT3	This study
AVL_J331	Diseased *Dicentrarchus labrax*, Spain	This study
AVL_J331CRD	AVL_J331 cured of pPHDPT3	This study
SSS256	PP3 ∆*vcrD^1^::cat*	This study
SSS325	PP3 ∆*vcrD^2^::cat*	This study
SSS299	PP3 ∆*vcrD^1^::cat* ∆*vcrD^2^::kan*	This study
SSS260	PP3 cured of pPHDPT3	This study
*Escherichia coli*		
DH5α	Cloning strain	Laboratory stock
S17-1-λpir	RP4-2 (Kan::Tn*7*,Tc::Mu-1) *pro-82* λ*pir recA1 endA1 thiE1 hsdR17 creC510*	[64]
β3914	RP4-2-Tc::Mu *∆dapA*::(*erm*-*pir*) *gyrA462 zei-298*::Tn*10* (Kan^R^ Em^R^ Tc^R^)	[65]
Plasmids		
pNidKan	Suicide vector, pCVD442 derivative, Kan^R^	[66]
pKD3	Template for *cat* gene amplification (Cm^R^)	[67]
pKD4	Template for *kan* gene amplification (Kan^R^)	[67]

**Table 5:** Oligonucleotides used in this study.

Oligonucleotides	Sequence (5′-3′) ^a^	Size (bp)
Tetracycline efflux pump *tetB*		
3′_int_tetB	TGAAGTGGTTCGGTTGGTTA	319
5′_int_tetB	AATAGCACCCACACCGTTGC	
Tetracycline-resistance ribosomal protection protein *tetM*		
5′_int_tetM	TTCAACAGCCGTTTGCAGCA	247
3′_int_tetM	TCTTTATAGTGGCGTACTGC	
*aip56* toxin gene (pPHDP10 plasmid)		
aip56_F	TCACGTTACAGGCTCTAGTG	388
aip56_R	GCATTCAACTGAACTGTCGG	
*frpA* piscibactin receptor gene (pPHDP70 plasmid)		
frpA_5′	GTGGTGTCACTTACAGCGAT	229
frpA_3′	GAGACAGAAAACGTCACAGC	
*parAB* partition genes (pPHDPT3 plasmid)		
parA_PP3_F	TCGTTCGTTTGGAGAATGGC	582
parB_PP3_R	GCCAATCGCAGGGTAGAACT	
T3SS gene *vscJ* (pPHDPT3 plasmid)		
vscJ_PP3_F	GCGGAGACGAAATCAGATCG	239
vscJ_PP3_R	TCAGGCCGAACTTTACACCG	
T3SS gene *vopD* (pPHDPT3 plasmid)		
vopD_PP3_F	GTAATACCTGCAAGCACACC	322
vopD_PP3_R	CAATCGGCGATCAAGCTAGA	
T3SS gene *yopN* (pPHDPT3 plasmid)		
yopN_PP3_F	GCGCTCAACCACATCCTTGT	431
yopN_PP3_R	AAGCGCATGAGCTGGTTTCC	
T3SS gene *vscQ* (pPHDPT3 plasmid)		
vscQ_F	GGCGTTGCTCAGTAGCCAAA	363
vscQ_R	GAGACCTGAAGCAGCACTTG	
T3SS gene *vopD* (pPHDPT3 plasmid)		
vopD_F	GTAATACCTGCAAGCACACC	
vopD_R	TCACTATCGCTGCTTACACT	185
*vcrD* delection		
vcrD_Xhol_1	GCCTCGAGGAGAAGAGCCATCAGTACCT	2041
vcrD_BamHI_2	GCGGATCCTTCTAGCGGTGTGGTGATGT	
vcrD_BamHI_3	GCGGATCCTTAGCGCTTGACCCTTCTGT	2027
vcrD_NotI_4	GCGCGGCCGCACTTGAACATCCGCTAAGCC	
vcrD_int_F	ATGCTTGCCGTGATGCTACT	1943
vcrD_int_R	GACACCACCAGTACAGGTTT	
Kanamycin resistance gene from pKD4 plasmid (*kan*)		
kanR_pKD4_BamHI_5′	GCGGATCCTAGAAAGCCAGTCCGCAGAA	1190
kanR_pKD4_BamHI_3′	GCGGATCCGAAGCCCAACCTTTCATAGA	
Chloramphenicol resistance gene from pKD3 plasmid (*cat*)		
cat_pKD3_BamHI_5′	GCGGATCCTACCTGTGACGGAAGATCAC	882
cat_pKD3_BamHI_3′	GCGGATCCGGAACTTCATTTAAATGGCG	

^a^ Underlined letters indicate the target of restriction enzymes.

## Data Availability

The PacBio-derived sequence of the Pdp PP3 genome was deposited in GenBank database under Accession number SRHT00000000.

## Supplementary Materials

The following supporting information can be downloaded at: https://www.mdpi.com/article/10.3390/ijms23094729/s1.
