# Supplementary material for: A Highly Unstable and Elusive Plasmid That Encodes the Type III Secretion System Is Necessary for Full Virulence in the Marine Fish Pathogen Photobacterium damselae subsp. piscicida
Source: Int J Mol Sci. 2022 Apr 25;23(9):4729. doi: 10.3390/ijms23094729 (PMC9105992; doi:10.3390/ijms23094729)
Supplement: Supplementary file 1 [file ijms-23-04729-s001.zip › Supplementary material_Figures.pdf]

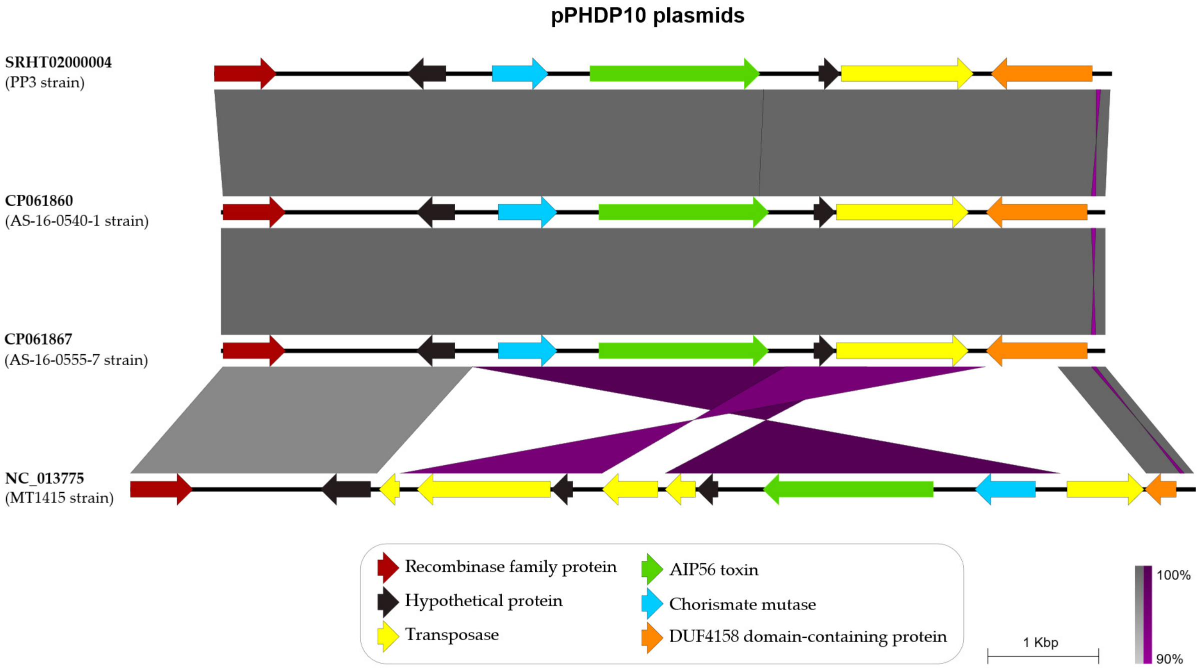

Figure S1: Comparative analysis of pPHDP10 plasmids from *Pdp* strains. Open reading frames (ORFs) are shown as arrows to indicate the direction of transcription and are colored in accordance with their predicted gene functions. Homologous segments (representing ≥90% sequence identity) are indicated by light gray shading. Inversions between AS-16-0555-7 and MT1415 strains are marked in purple. Regions are drawn to scale from accession numbers SRHT02000004 (PP3 strain), CP061860 (AS-16-0540-1 strain), CP061867 (AS-16-0555-7 strain) and NC_013775 (MT1415 strain). The alignment is a pairwise BLASTn alignment performed using Easyfig.

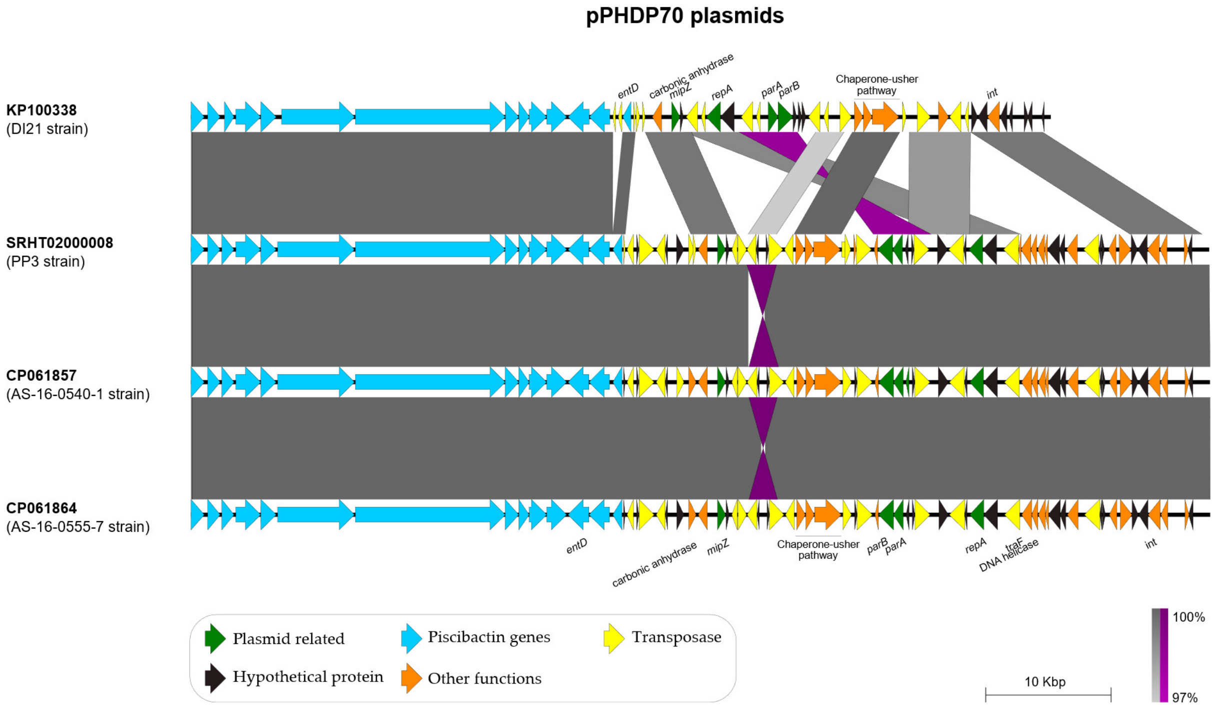

Figure S2: Comparative analysis of pPHDP70 plasmids in *Pdp* strains. The complete sequence of pPHDP70 from strain DI21 was used as a reference. Open reading frames (ORFs) are shown as arrows to indicate the direction of transcription and are colored in accordance with their predicted gene functions. Homologous segments (representing ≥97% sequence identity) are indicated by light gray shading. Inversions between strains are marked in purple. Regions are drawn to scale from accession numbers KP100338 (DI21 strain), SRHT02000008 (PP3 strain), CP061857 (AS-16-0540-1 strain) and CP061864 (AS-16-0555-7 strain). The alignment is a pairwise BLASTn alignment performed using Easyfig.

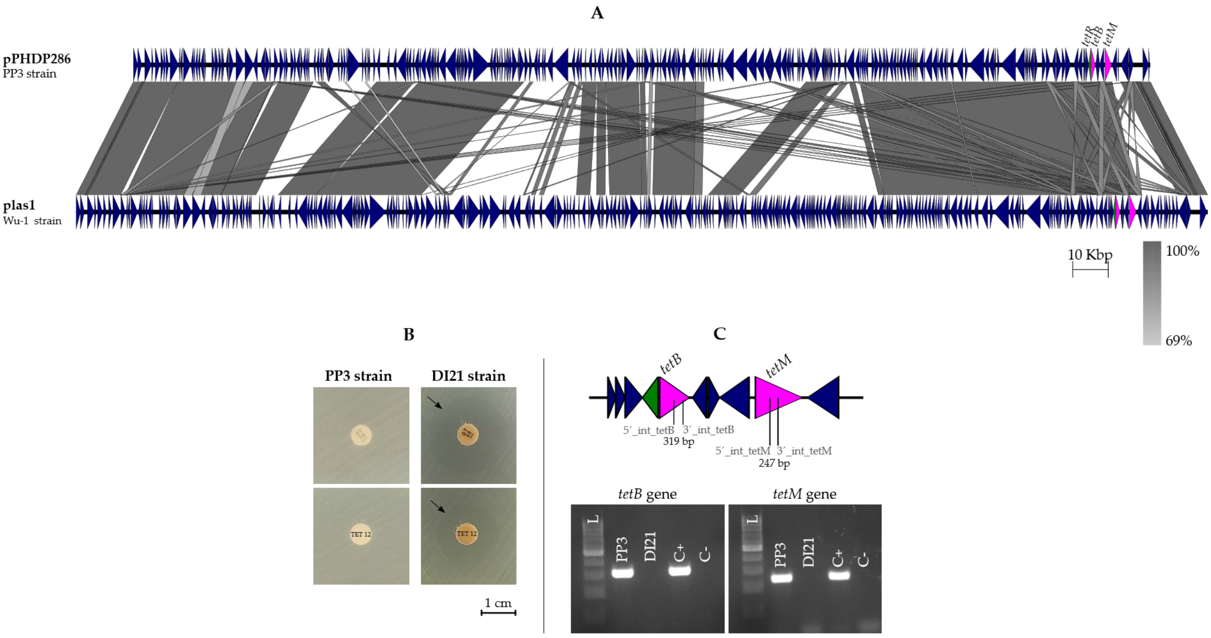

Figure S3: (A) Comparison between pPHDP286 and plas1, two plasmids harbouring tetracycline resistance genes in *Pdp* PP3 and *Pdd* Wu-1 strains, respectively. Open reading frames (ORFs) are shown in dark blue with the exception of genes related to tetracycline resistance that are highlighted in green and magenta. Homologous segments (representing ≥69% sequence identity) are indicated by light gray shading. Regions are drawn to scale from accession numbers SRHT02000003 (pPHDP286 plasmid of *Pdp* PP3) and NZ_CP018299 (plas1 plasmid of *Pdd* Wu-1). (**B**) Antibiogram of *Pdp* PP3 strain harbouring *tetB* and *tetM* and of the non-carrier strain *Pdp* DI21. The arrows denote the extent of the sensitivity haloes. Bar, 1 cm. (**C**) Amplification of *tetB* and *tetM* genes in tetracycline-resistant strain PP3 and absence of amplification in sensitive strain DI21. C+: positive control; C-: negative control; L: GeneRuler 1 kb DNA Ladder (Thermo Scientific™).

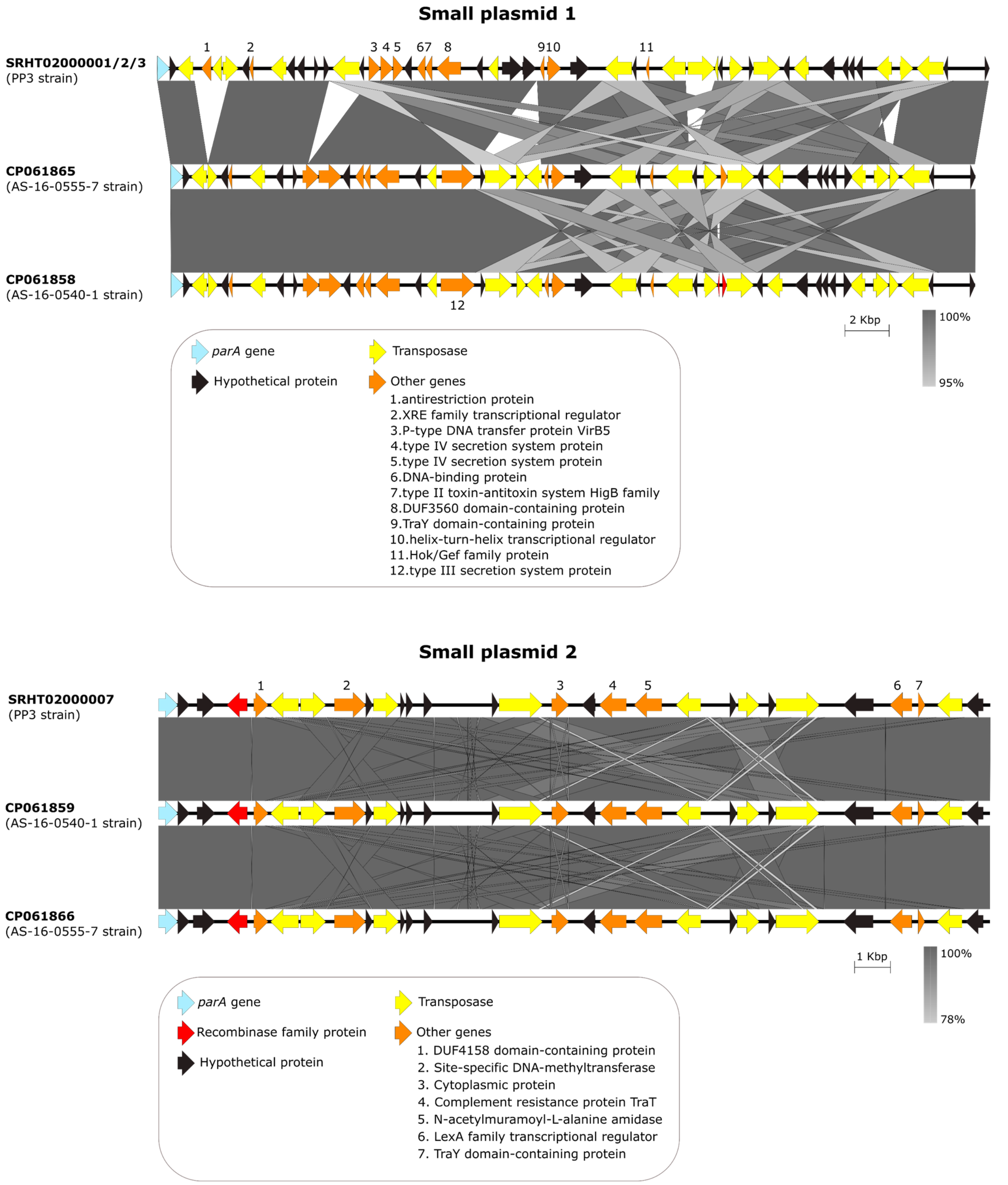

Figure S4: Comparative analysis of *small plasmid 1* and *small plasmid 2* from *Pdp* strains. Open reading frames (ORFs) are shown as arrows to indicate the direction of transcription and are colored in accordance with their predicted gene functions. Homologous segments indicated by light gray shading. Regions were drawn to scale from accession numbers indicated in the figure. The alignment is a pairwise BLASTn alignment performed using Easyfig.

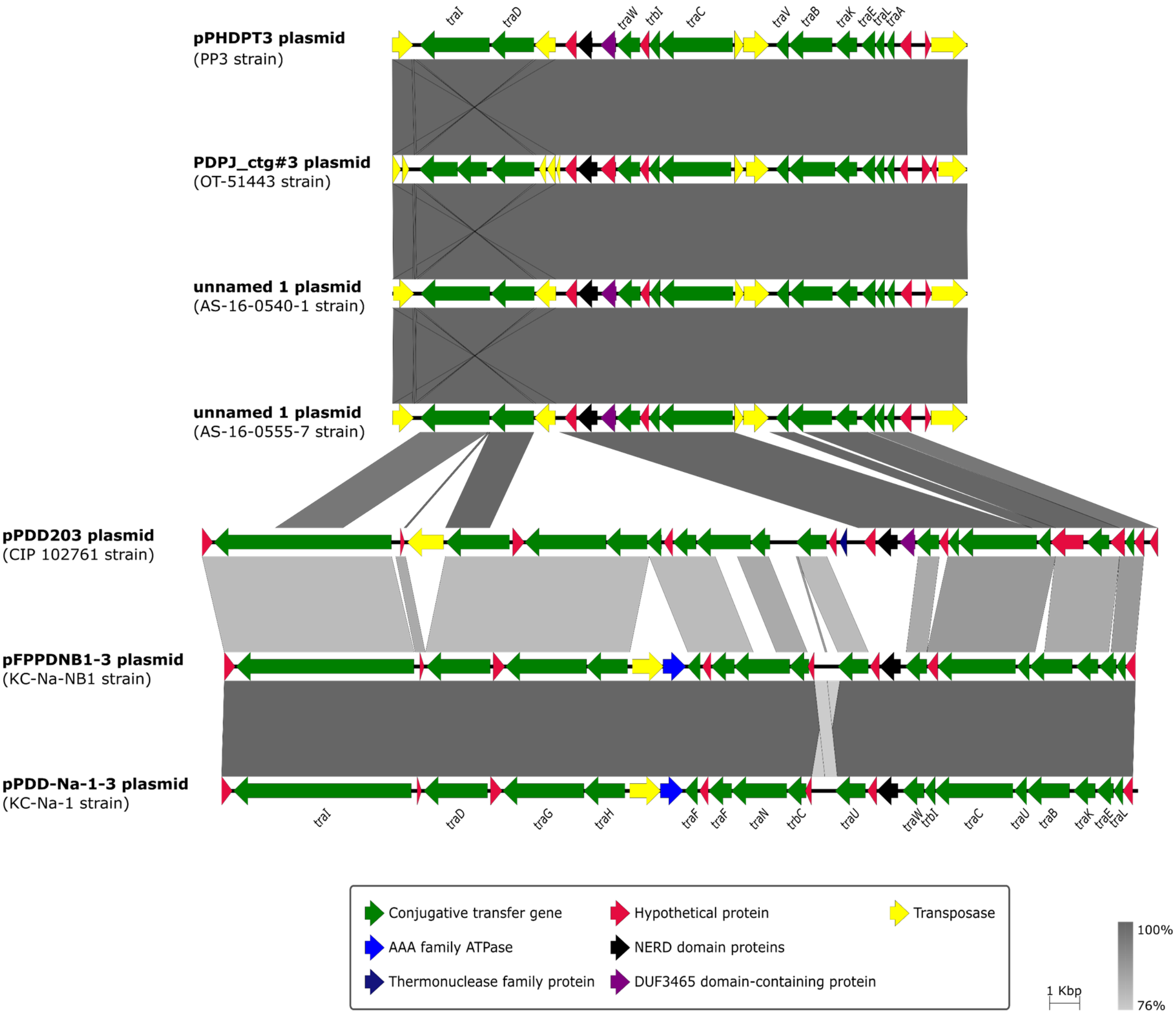

Figure S5: Linear genetic representation of the conjugation genes region in *P. damselae* plasmids: pPHDPT3 of *Pdp* PP3 (SRHT02000010); contig 3 of *Pdp* OT-51443 (BDMQ01000003); unnamed 1 plasmid of *Pdp* AS-16-0540-1 (CP061856); unnamed 1 plasmid of *Pdp* AS-16-0555-7 (CP061863); pPHDP203 of *Pdd* CIP 102761 (NZ_ADBS01000003); pFPPDNB1-3 of *Pdd* KC-Na-NB1 (CP035461 NZ_CP035461) and pPDD-Na-1-3 of *Pdd* KC-Na-NB1 (NZ_CP021155). The arrows depict the open reading frame organization within each cluster and the orientation of the arrows indicates direction of transcription. Grey-shaded vertical blocks connect homologous sequences, the color intensity indicating the identity levels (from 76% to 100%).

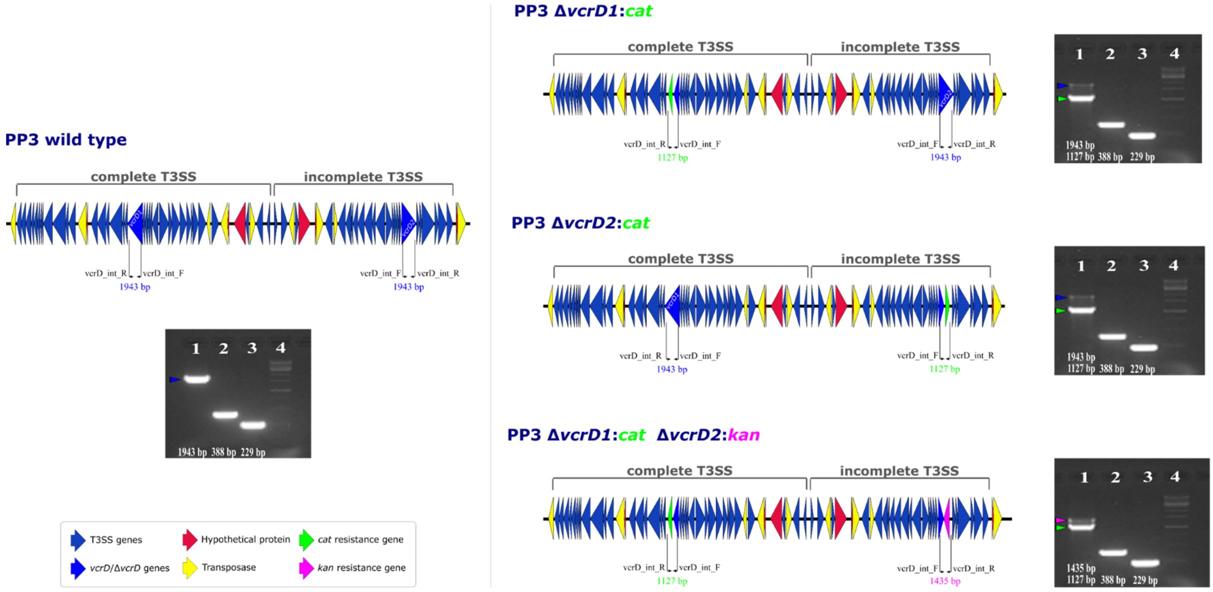

Figure S6: Schematic illustration to show the *vcrD*[1] and *vcrD*[2] gene location within the T3SS gene clusters and the strategy to generate single and double *vcrD* gene mutants by insertion of antibiotic resistance cassettes into the deleted versions of *vcrD*. Demonstration of *vcrD* alleles disruption was conducted with PCR amplifications using specific primer combinations as described in Table 6. Gene markers for pPHDP10 and pPHDP70 were amplified as controls, to verify the stability of these virulence plasmids. Agarose gel lanes are labeled as follows: (1) pPHDPT3_*vcrD* gene: 1,943 bp, (2) pPHDP10_*aip56* gene: 388 bp, (3) pPHDP70_*frpA* gene: 229 bp, and GeneRuler 1 kb DNA Ladder (Thermo Scientific™). The arrows at the left side of the agarose gels indicate the PCR amplicons for either the wild type or deletion-mutant versions of each *vcrD* allele. The green arrows indicate the chloramphenicol cassette location and orientation, and the magenta arrows indicate the kanamycin cassette location and orientation.
